# Effects of Tail Pinch on BDNF and trkB Expression in the Hippocampus of Roman Low- (RLA) and High-Avoidance (RHA) Rats

**DOI:** 10.3390/ijms24119498

**Published:** 2023-05-30

**Authors:** Maria Pina Serra, Francesco Sanna, Marianna Boi, Marcello Trucas, Alberto Fernández-Teruel, Maria Giuseppa Corda, Osvaldo Giorgi, Marina Quartu

**Affiliations:** 1 Department of Biomedical Sciences, Section of Cytomorphology, University of Cagliari, Cittadella Universitaria di Monserrato, 09042 Monserrato, Italymarianna.boi@unica.it (M.B.); marcello.trucas@unica.it (M.T.); 2 Department of Life and Environmental Sciences, Section of Pharmaceutical, Pharmacological and Nutraceutical Sciences, University of Cagliari, Cittadella Universitaria di Monserrato, 09042 Monserrato, CA, Italy; 3 Medical Psychology Unit, Department of Psychiatry and Legal Medicine, Institute of Neuroscience, School of Medicine, Autonomous University of Barcelona, 08193 Barcelona, Spain

**Keywords:** BDNF, trkB, tail pinch, stress, depression, Roman high- and low-avoidance rats, hippocampus, Western blot, immunohistochemistry

## Abstract

In this article, we describe the effects of tail pinch (TP), a mild acute stressor, on the levels of brain-derived neurotrophic factor (BDNF) and its tyrosine kinase receptor B (trkB) proteins in the hippocampus (HC) of the outbred Roman High- (RHA) and Low-Avoidance (RLA) rats, one of the most validated genetic models for the study of fear/anxiety- and stress-related behaviors. Using Western blot (WB) and immunohistochemistry assays, we show for the first time that TP induces distinct changes in the levels of BDNF and trkB proteins in the dorsal (dHC) and ventral (vHC) HC of RHA and RLA rats. The WB assays showed that TP increases BDNF and trkB levels in the dHC of both lines but induces opposite changes in the vHC, decreasing BDNF levels in RHA rats and trkB levels in RLA rats. These results suggest that TP may enhance plastic events in the dHC and hinder them in the vHC. Immunohistochemical assays, carried out in parallel to assess the location of changes revealed by the WB, showed that, in the dHC, TP increases BDNF-like immunoreactivity (LI) in the CA2 sector of the Ammon’s horn of both Roman lines and in the CA3 sector of the Ammon’s horn of RLA rats while, in the dentate gyrus (DG), TP increases trkB-LI in RHA rats. In contrast, in the vHC, TP elicits only a few changes, represented by decreases of BDNF- and trkB-LI in the CA1 sector of the Ammon’s horn of RHA rats. These results support the view that the genotypic/phenotypic features of the experimental subjects influence the effects of an acute stressor, even as mild as TP, on the basal BDNF/trkB signaling, leading to different changes in the dorsal and ventral subdivisions of the HC.

## 1. Introduction

The Roman low- (RLA) and high-avoidance (RHA) rats were psychogenetically selected for poor vs. rapid acquisition of two-way active avoidance [1,2] and represent two divergent phenotypes that, in the face of stressors, display reactive (RLA) vs. proactive (RHA) coping styles [3,4]. Remarkably, the higher fearfulness/anxiety of RLA rats compared to their RHA counterparts is also related to a stress-induced depression-like phenotype that returns to normal upon chronic antidepressant treatment [5,6], thus supporting the concept that both environmental stressors and the genetic background have a central role in the etiopathology of depression. 

Preclinical and clinical studies of gene expression and imaging sustain the hypothesis that depression and the response to antidepressant drugs are regulated by neurotrophic factors. In particular, the neurotrophic hypothesis of depression posits that decreased cell support mediated by the brain-derived neurotrophic factor (BDNF) and its tyrosine-protein kinase B (trkB) receptor leads to decreased neurogenesis, neuronal atrophy, and glial cell loss in the hippocampus (HC) and that the BNDF deficit is blocked or reversed by antidepressant drug treatment [7,8]. 

Because depression is often triggered or worsened by acute or chronic stressful life events, and stress can affect neuronal integrity and viability in specific brain structures [7,9], most preclinical models of depression are based on stress-inducing behavioral paradigms to study alterations of the brain structure and function. Importantly, a decline in the levels of the BDNF mRNA and/or protein and signaling in the HC and prefrontal cortex (PFC) are frequent features of several stress paradigms [10,11,12]. Accordingly, we have previously reported that the basal BDNF levels are lower in the HC of RLA vs. RHA rats [13] and, more recently, we have shown that the basal BDNF- and trkB levels are lower in the PFC of RLA vs. RHA rats [14]. Together, these findings suggest that the basal BDNF/trkB signaling is weaker in the HC and PFC of rats prone to developing a stress-induced depression-like phenotype compared to depression-resilient rats.

It is noteworthy, however, that the cellular and molecular underpinnings of BDNF’s involvement in the response to stress in animal models and the origin and development of depression remain unclear. The intensity of the stressor may impact distinctly on the expression of BDNF and its tyrosine-protein kinase B (trkB) receptor in different brain areas involved in driving reward- and emotion-related behaviors. For instance, repeated social defeat stress elicits dynamic alterations of BDNF expression, causing both short-term increases in the PFC and long-term upregulation in the medial amygdala and ventral tegmental area (VTA) [15]. Conversely, in animals displaying a stress-induced depression-like phenotype, a single bilateral infusion of BDNF in the HC mimics the normalization of the depression-like behavior elicited by antidepressant drugs [16]. The local infusion of BDNF in the VTA, instead, elicits a depression-like decrease in social interaction [17]. These findings suggest opposing effects of BDNF, acting as an antidepressant in the HC and as a pro-depressant in the VTA, supporting the view that different molecular mechanisms and neuronal pathways are involved in the effects of BDNF in the etiopathology of depression. 

In keeping with the above findings, our previous study using Western blot and immunohistochemistry assays has addressed the association between the dynamic rapid changes of BDNF in the dorsal and ventral subdivisions of the HC and the exposure to an intense acute stressor such as forced swimming (FS) in cold water (24–25 °C) for 15 min [18]. Thus, our results showed that, in RLA rats, the FS causes a marked decrease in BDNF protein levels in the ventral HC (vHC) associated with a significant increase in the dorsal HC (dHC). In contrast, in RHA rats, notwithstanding the decrease in BDNF-like immunoreactivity in the vHC subregions, no significant changes in BDNF protein levels were observed in either hippocampal compartment of stressed vs. control rats [18]. Hence the evidence obtained so far is consistent with the view that distinct alterations in the levels of BDNF are under the influence of multiple factors, such as the brain area examined, the modality of the stressor, and the genetic background of the experimental subjects. 

Because, as mentioned above, the intensity of the stressor may impact differently on BDNF expression in distinct brain areas, it was considered of interest to examine the effects of a mild stressor on BDNF/trkB signaling in the HC of the Roman lines. To this aim, we investigated the impact of a very mild and arousal-inducing stressor like tail pinching (TP) [19,20,21,22] for 40 min on the levels of BDNF and trkB proteins in the brain of RLA and RHA rats carrying out Western blots of the dHC and vHC and immunohistochemistry (IHC) assays in the CA1, CA2, CA3 sectors of the Ammon’s horn, and dentate gyrus (DG) hippocampal subfields. 

## 2. Results

### 2.1. Behavioral Measures during TP

In keeping with previous studies [4], when submitted to TP for 40 min, RHA rats displayed intense proactive coping behavior while RLA rats exhibited a reactive coping activity. Thus, RHA rats spent a longer time biting the clamp to remove it from their tails (*p* < 0.0001), whereas the performance of RLA rats was characterized by spending significantly longer times licking their tails (*p* < 0.001), freezing (*p* < 0.0001) and grooming (*p* < 0.05) than RHA rats (Table 1).

### 2.2. Western Blot

#### 2.2.1. BDNF Protein Levels

**Figure 1:**
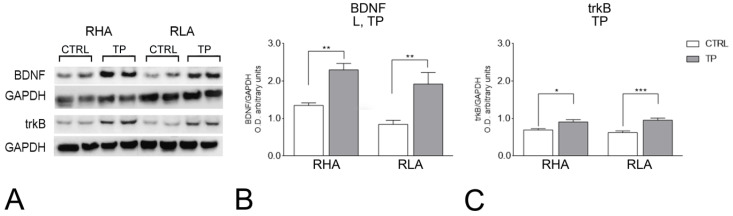
Western blot analysis of the brain-derived neurotrophic factor (BDNF) (**A**,**B**) and trkB (**A**,**C**) in the dorsal hippocampus of RHA and RLA rats, under basal conditions (CTRL), and after tail pinch (TP). (**A**): BDNF-, trkB-, and GAPDH-immunostained blots showing representative samples from two rats; (**B**,**C**): Densitometric analysis of the BDNF/GAPDH (**B**) and trkB/GAPDH (**C**) band gray optical density (O.D.) ratios. Columns and bars denote the mean ± S.E.M. of eight rats in each experimental group. *: *p* < 0.05; **: *p* < 0.01; ***: *p* < 0.001. (Tukey’s *post-hoc* test for multiple comparisons).

**Figure 2:**
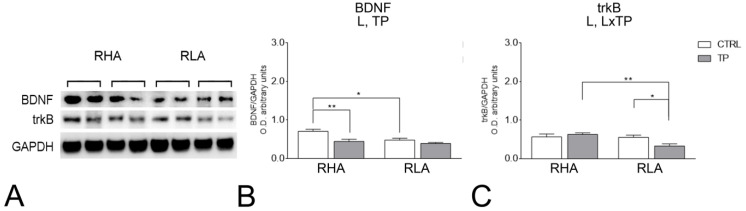
Western blot analysis of the brain-derived neurotrophic factor (BDNF) (**A**,**B**) and trkB (**A**,**C**) in the ventral hippocampus of RHA and RLA rats, under basal conditions (CTRL), and after tail pinch (TP). (**A**): BDNF-, trkB-, and GAPDH-immunostained blots showing representative samples from two rats; (**B**,**C**): Densitometric analysis of the BDNF/GAPDH (**B**) and trkB/GAPDH (**C**) band gray optical density (O.D.) ratios. Columns and bars denote the mean ± S.E.M. of eight rats in each experimental group. *: *p* < 0.05; **: *p* < 0.001. (Tukey’s *post-hoc* test for multiple comparisons).

The antibody against BDNF recognized a protein band with a relative molecular weight (mw) ≅ 13 kDa (Figure 1 and Figure 2), in agreement with the reported mw of the monomeric form of the protein [23]. The statistical evaluation with two-way ANOVA (main factors: rat line and treatment, i.e., TP) of the densitometric values of BDNF-like immunoreactivity (LI) in tissue homogenates from the dHC revealed significant effects of line and treatment (*p* values < 0.05; Table S1); subsequent pairwise *post-hoc* contrasts showed that, in both RHAs and RLAs, TP increased the BDNF-LI by 70% (*p* = 0.0072) and 127% (*p* = 0.0021) over the respective control group (Figure 1B). In the vHC, a two-way ANOVA revealed significant effects of line and treatment (*p* values < 0.05; Table S2) and *post-hoc* contrasts showed that the relative baseline level of BDNF-LI of the RLAs was 31% (*p* = 0.0316) lower than that of their RHA counterparts. Additional pairwise contrasts showed that, after TP, the relative level of the BDNF-LI of the RHA rats was 37% (*p* = 0.0072) lower than that of the respective control (Figure 2B). 

#### 2.2.2. trkB Protein Levels

The antibody against the full-length form of trkB recognized a protein band with a relative mw ≅ 140 kDa (Figure 1 and Figure 2), consistent with the reported mw of the receptor protein [24]. The statistical evaluation with two-way ANOVA (main factors: rat line and treatment, i.e., TP) of the densitometric values of trkB-LI in tissue homogenates from the dHC revealed a significant interaction line × TP (*p* value < 0.0001; Table S1) and subsequent pairwise contrasts showed that, in the RHAs and RLAs, TP increased the trkB-LI by 31% (*p* = 0.0367) and 53% (*p* = 0.0007) over the respective control group (Figure 1C). In the vHC, a two-way ANOVA revealed a significant effect of line and a significant interaction line x treatment (*p* values < 0.05; Table S1) and *post-hoc* contrasts showed that, after TP, the relative level of trkB-LI of RLA rats was 41% lower (*p* = 0.0394) than the respective control (Figure 2C). Further pairwise contrasts showed that, after TP, the relative level of trkB-LI of the RLAs was 48% (*p* = 0.0056) lower than that of their RHA counterparts (Figure 2). 

### 2.3. Immunohistochemistry

**Figure 3:**
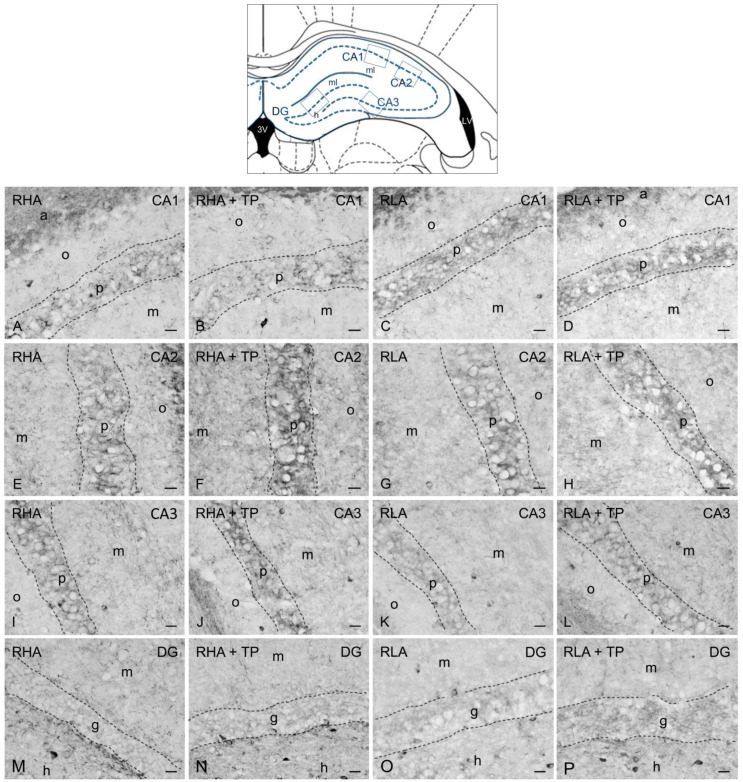
Brain-derived neurotrophic factor-like immunoreactivity in the dorsal hippocampus of RHA (first and second column) and RLA rats (third and fourth column) under basal conditions and after tail pinch (TP). Upper panel: schematic representation of the dorsal hippocampus (Figure 33, modified from Paxinos and Watson [25]); the boxes superimposed over the outline denote the microscopic fields shown at higher magnification in (**A**–**P**). (**A**–**D**): CA1 sector; (**E**–**H**): CA2 sector; (**I**–**L**): CA3 sector of the Ammon’s horn; (**M**–**P**): dentate gyrus (DG). The dashed lines mark the boundaries of the Ammon’s horn pyramidal layer (p) and DG granule cell layer (g). a, alveus; h, hilus; m, molecular layer; o, stratum oriens. Scale bars: 25 μm.

**Figure 4:**
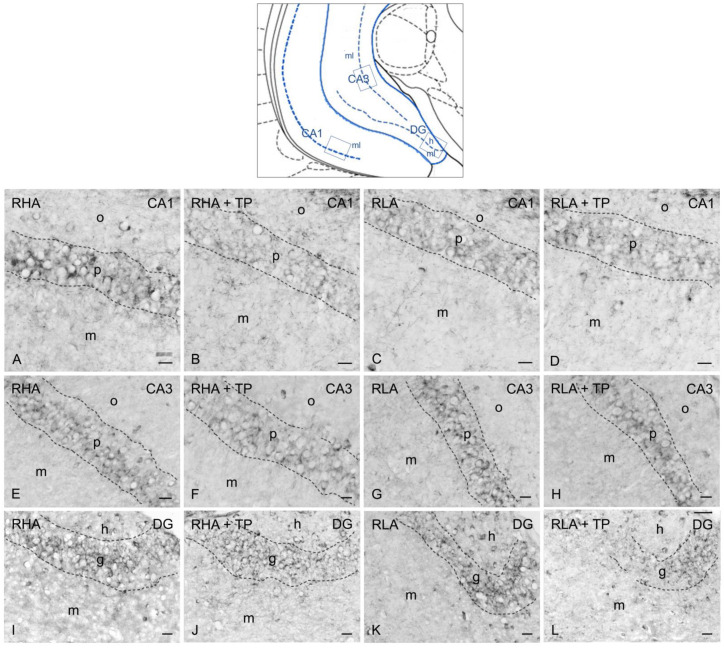
Brain-derived neurotrophic factor-like immunoreactivity in the ventral hippocampus of RHA (first and second column) and RLA rats (third and fourth column) under basal conditions and after tail pinch (TP). Upper panel: schematic representation of the ventral hippocampus (Figure 44, modified from Paxinos and Watson [25]); the boxes superimposed over the outline denote the microscopic fields shown at higher magnification in (**A**–**L**). (**A**–**D**): CA1 sector; (**E**–**H**): CA3 sector of the Ammon’s horn; (**I**–**L**): dentate gyrus (DG). The dashed lines mark the boundaries of the Ammon’s horn pyramidal layer (p) and DG granule cell layer (g). h, hilus; m, molecular layer; o, stratum oriens. Scale bars: 25 μm.

**Figure 5:**
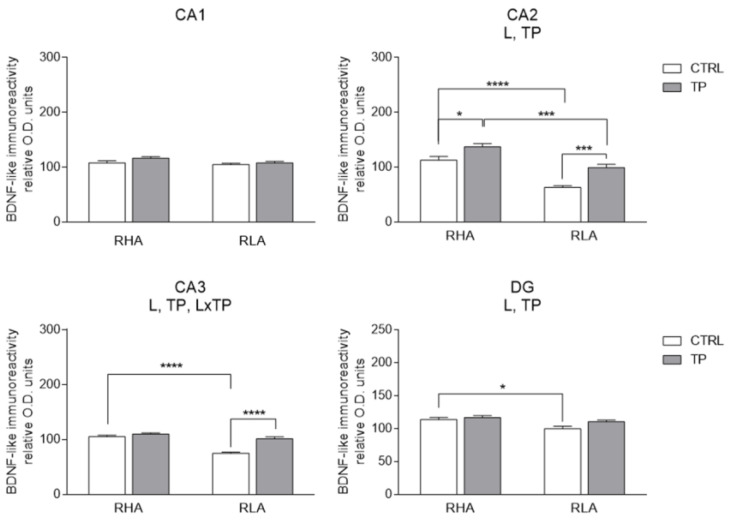
Densitometric analysis of brain-derived neurotrophic factor (BDNF)-like immunoreactivity in the CA1–CA3 sectors of the Ammon’s horn and the dentate gyrus (DG) of the dorsal hippocampus under basal conditions (CTRL) and after tail pinch (TP). Columns and bars denote the mean ± S.E.M. of six rats in each experimental group. Two different sections were analyzed for each rat. *: *p* < 0.05; ***: *p* < 0.001; ****: *p* < 0.0001. (Tukey’s *post-hoc* test or Sidak’s correction for multiple comparisons).

**Figure 6:**
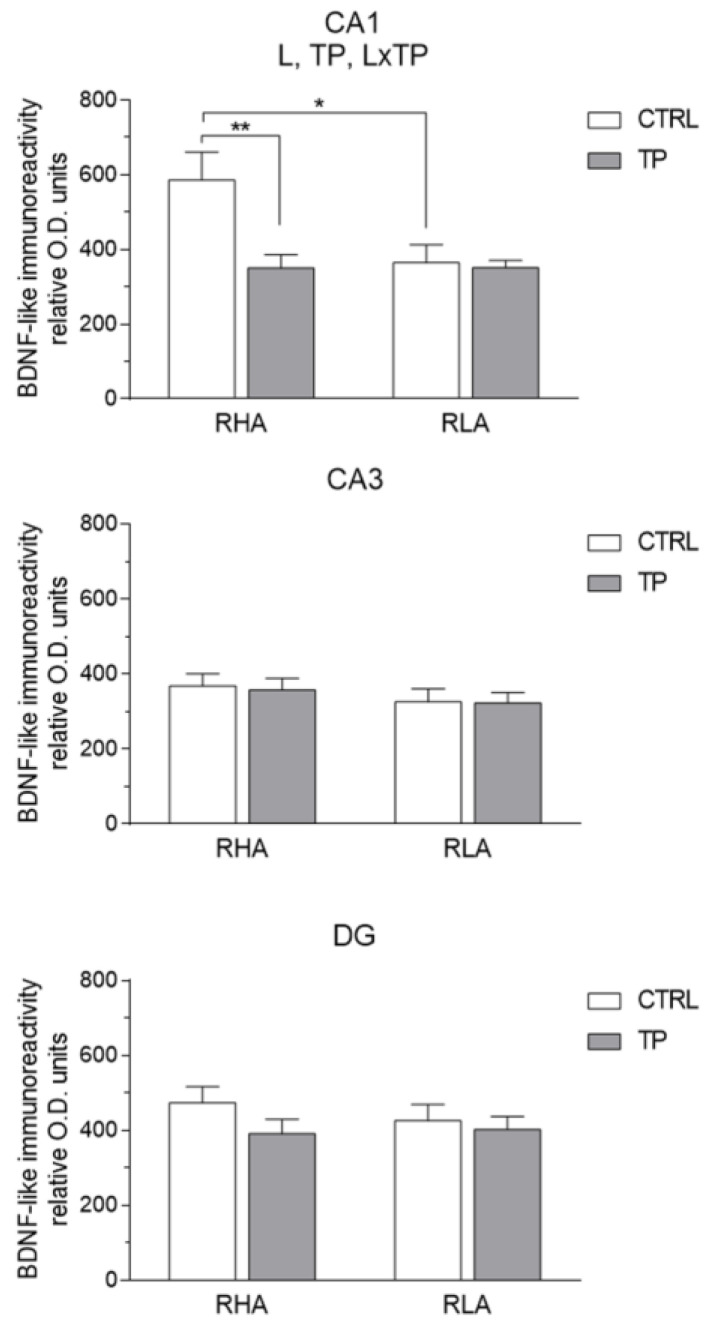
Densitometric analysis of brain-derived neurotrophic factor (BDNF)-like immunoreactivity in the CA1 and CA3 sectors of the Ammon’s horn and the dentate gyrus (DG) of the ventral hippocampus under basal conditions (CTRL) and after tail pinch (TP). Columns and bars denote the mean ± S.E.M. of six rats in each experimental group. Two different sections were analyzed for each rat. *: *p* < 0.05; **: *p* < 0.01. (Tukey’s *post-hoc* test or Sidak’s correction for multiple comparisons).

As previously described [13,14], the BDNF- (Figure 3 and Figure 4) and trkB-immunostained structures (Figure 5 and Figure 6) were unevenly distributed within the hippocampal formation and represented by labeled cell bodies, neuronal proximal processes, and nerve fibers in the Ammon’s horn and the dentate gyrus. BDNF- and trkB-labeled nerve fibers were also observed in the alveus and the fimbria.

#### 2.3.1. BDNF-like Immunoreactivity

The majority of BDNF-like immunoreactive fiber networks were distributed in the Ammon’s horn (Figure 3 and Figure 4) where the labeling had mostly the aspect of filamentous elements running in between the perikarya of the pyramidal layer and in the molecular layers of the CA1 (Figure 3A–D and Figure 4A–D), CA2 (Figure 3E–H), and CA3 sectors of the Ammon’s horn (Figure 3I–L and Figure 4E–H). BDNF-labeled neuronal cells were also observed in the pyramidal, molecular, and oriens layers (Figure 3I–K). Under basal conditions, the BDNF-like immunoreactive elements appeared to be generally denser in RHAs than RLAs. In the dentate gyrus, the BDNF-LI labeled neuronal perikarya in the granule cell layer, at the interface between the granule cell layer and the polymorphic layer, and in the hilus (Figure 3M–P and Figure 4I–L). BDNF-like immunoreactive nerve fibers appeared as loose meshes and punctate elements distributed in the molecular layer (Figure 3M–P) and in the hilus (Figure 3M–P and Figure 4I–L). 

The densitometric analysis in the CA sectors of the hippocampus proper and the dentate gyrus (Figure 4 and Figure 5) revealed significant differences in the BDNF-LI between the RHA and RLA lines, between the basal and TP conditions, and between the dHC and vHC. Thus, as shown in Table S2, in the dHC, the two-way ANOVA revealed effects of line and TP in the CA2 and CA3 sectors and in the DG, and a significant line × TP interaction in the CA3 sector. Moreover, pairwise contrasts showed that in the CA2 and CA3 sectors and the DG the basal BDNF-LI was significantly lower (−44%; *p* < 0.0001, −29%; *p* < 0.0001, and −12%; *p* = 0.0129, respectively) in RLA vs. RHA rats. After TP, the BDNF-LI was 22% (*p* = 0.0171) higher than the control value in the CA2 of RHAs, and 36% (*p* = 0.0003) and 35% (*p* < 0.0001) higher than the respective controls in the CA2 and CA3 sectors of RLAs (Figure 5). Additional pairwise contrasts showed that, upon TP, BDNF-LI in the CA2 sector was significantly lower in RLA than RHA rats (−38%; *p* = 0.0001) (Figure 5). In the vHC, a two-way ANOVA revealed significant effects of line and TP, as well as a line × TP interaction in the CA1 sector (Table S2). In addition, *post-hoc* contrasts showed that, in the CA1 sector, the basal BDNF-LI was 38% (*p* = 0.0125) lower in RLA vs. RHA rats and, upon TP, the BDNF-LI decreased by 40% (*p* = 0.0373) in the CA1 of RHA rats (Figure 6).

#### 2.3.2. trkB-like Immunoreactivity

**Figure 7:**
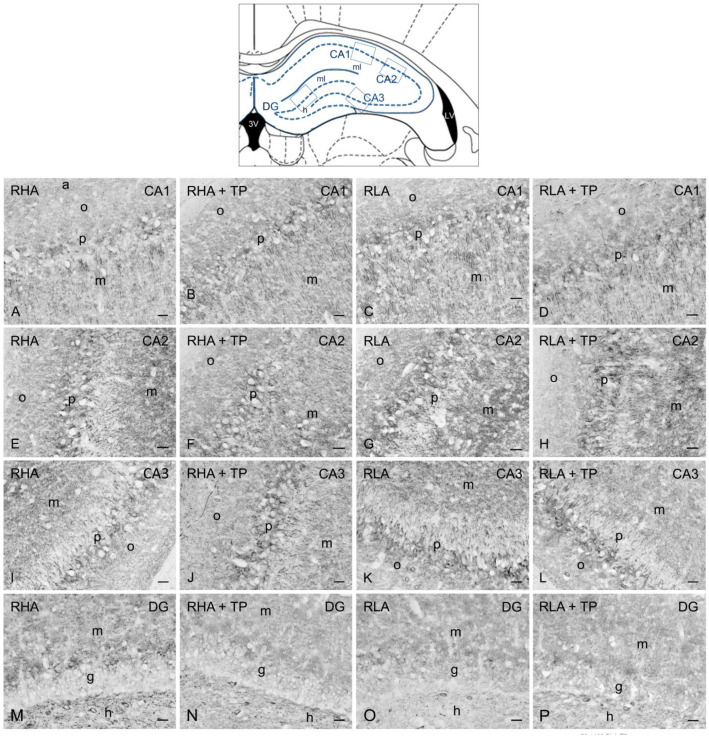
TrkB receptor-like immunoreactivity in the dorsal hippocampus of RHA (first and second column) and RLA rats (third and fourth column) under basal conditions and after tail pinch (TP). Upper panel: schematic representation of the dorsal hippocampus (Figure 33, modified from Paxinos and Watson [25]); the boxes superimposed over the outline denote the microscopic fields shown at higher magnification in (**A**–**P**). (**A**–**D**): CA1 sector; (**E**–**H**): CA2 sector; (**I**–**L**): CA3 sector of the Ammon’s horn; (**M**-**P**): dentate gyrus (DG). The dashed lines mark the boundaries of the Ammon’s horn pyramidal layer (p) and DG granule cell layer (g). h, hilus; m, molecular layer; o, stratum oriens. Scale bars: 25 μm.

**Figure 8:**
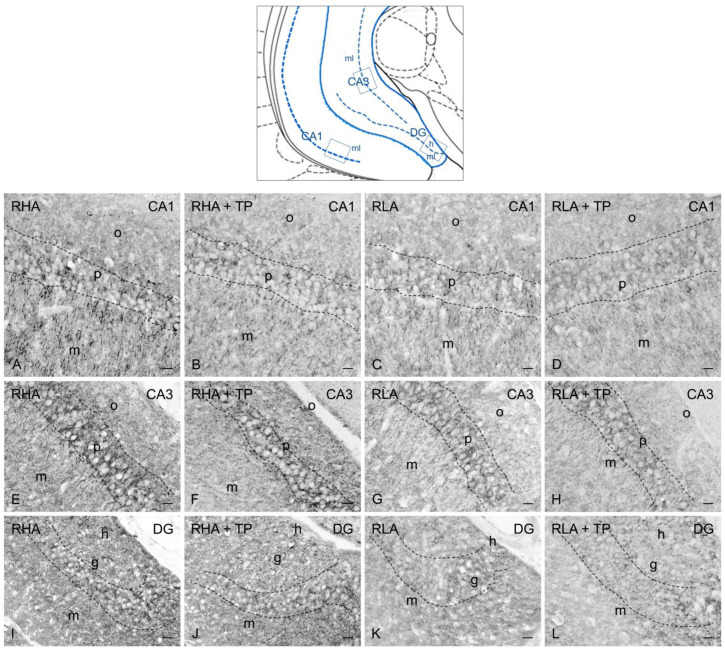
TrkB receptor-like immunoreactivity in the ventral hippocampus of RHA (first and second column) and RLA rats (third and fourth column) under basal conditions and after tail pinch (TP). Upper panel: schematic representation of the ventral hippocampus (Figure 44, modified from Paxinos and Watson [25]); the boxes superimposed over the outline denote the microscopic fields shown at higher magnification in (**A**–**L**). (**A**–**D**): CA1 sector; (**E**–**H**): CA3 sector of the Ammon’s horn; (**I**–**L**): dentate gyrus (DG). Dashed lines mark the boundaries of the Ammon’s horn pyramidal layer (p) and DG granule cell layer (g). h, hilus; m, molecular layer; o, stratum oriens. Scale bars: 25 μm.

The trkB-LI also labeled extensive nerve fiber systems, mostly appearing as filaments, short hollow tubules, and coarse dot-like elements (Figure 7 and Figure 8). In the Ammon’s horn, occasional trkB-immunolabeled neuronal perikarya or their proximal processes were observed (Figure 7A–L). In the DG, trkB-LI was localized to the filaments and dot-like structures distributed in the thickness of the granule cell layer, deep in the molecular layer, and in the hilus (Figure 7M–P). The trkB-labeled neuronal cell bodies were observed in the hilus (Figure 7M–P and Figure 8I–L). Overall, under basal conditions, trkB-LI appeared to be lower in RLA vs. RHA rats, particularly in the vHC (Figure 7M–P), while upon TP, a decrease in immunoreactivity vs. the respective controls was observed in the CA1 sector of the vHC of RHA rats (Figure 8A,B).

**Figure 9:**
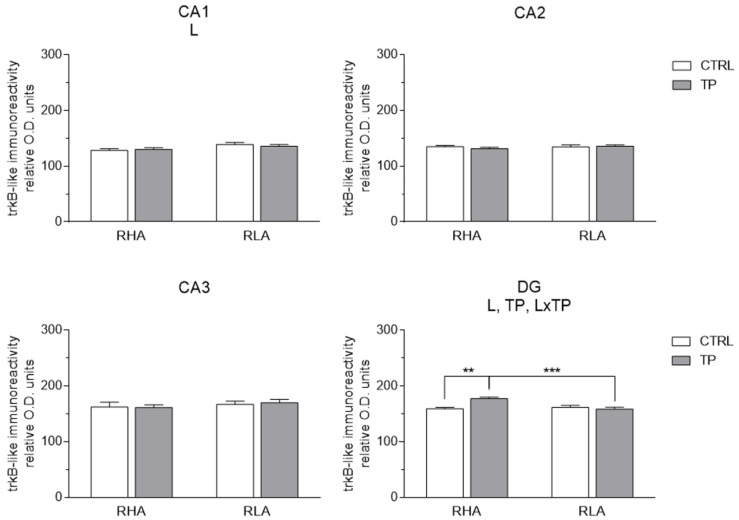
Densitometric analysis of trkB receptor-like immunoreactivity in the CA1–CA3 sectors of the Ammon’s horn and the dentate gyrus (DG) of the dorsal hippocampus under basal conditions (CTRL) and after tail pinch (TP). Columns and bars denote the mean ± S.E.M. of six rats in each experimental group. Two different sections were analyzed for each rat. **: *p* < 0.01; ***: *p* < 0.001. (Tukey’s *post-hoc* test or Sidak’s correction for multiple comparisons).

**Figure 10:**
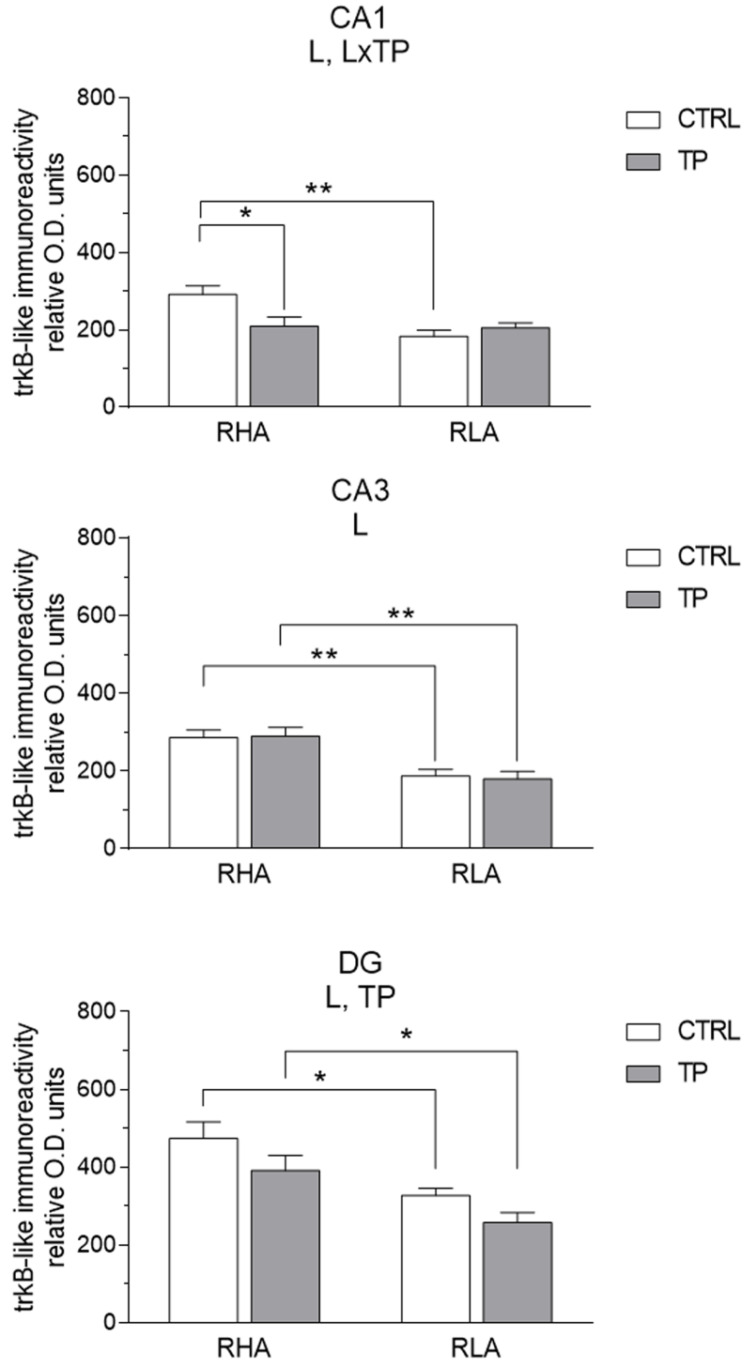
Densitometric analysis of trkB receptor-like immunoreactivity in the CA1 and CA3 sectors of the Ammon’s horn and the dentate gyrus (DG) of the ventral hippocampus under basal conditions (CTRL) and after tail pinch (TP). Columns and bars denote the mean ± S.E.M. of six rats in each experimental group. Two different sections were analyzed for each rat. *: *p* < 0.05; **: *p* < 0.01. (Tukey’s *post-hoc* test or Sidak’s correction for multiple comparisons).

The densitometric analysis in the Ammon’s horn and the DG (Figure 9 and Figure 10) revealed significant differences in the trkB-LI between the Roman lines, between the control and stressed rats, and between the dHC and vHC. In the dHC, the two-way ANOVA revealed an effect of line in the CA1 sector, and the effects of line, TP, and a line × TP interaction in the DG (Table S2). *Post-hoc* contrasts showed that, upon TP, the trkB-LI of RHA rats was 11% higher (*p* = 0.0014) than the respective control in the DG while the trkB-LI was lower (−12%; *p* = 0.0007) in RLA than RHA rats (Figure 9). In the vHC, the two-way ANOVA revealed a significant line effect in the CA1 and CA3 sectors and the DG, and a line × treatment interaction in the CA1 (Table S2). Moreover, *post-hoc* contrasts showed that in the CA1 and CA3 sectors and in the DG the basal trkB-LI was significantly lower (−37%; *p* = 0.0013, −34%; *p* = 0.005, and −31%; *p* = 0.0003, respectively) in RLA vs. RHA rats. Additional pairwise contrasts revealed that, after TP, trkB-LI was significantly decreased (−39%; *p* = 0.0207) in the CA1 of RHA rats; moreover, *post-hoc* contrasts showed that, upon TP, trkB-LI was significantly lower in the CA3 sector and the DG (−38%; *p* = 0.0016 and −34%; *p* = 0.037, respectively) of RLA than RHA rats (Figure 10).

## 3. Discussion

The Roman rat lines represent a model to investigate the impact of the interactions between genetic and aversive environmental factors on the neural substrates of depression [3,26]. The emotional reactivity, rather than cognitive processes, is the prominent behavioral difference between the two lines, with RLAs being more fearful/anxious in the face of aversive events than their RHA counterparts, who behave as proactive copers in response to novelty and stressful events [3,4,26]. 

The present results showed that when exposed to a 40 min TP session, a milder stressor compared to forced swimming (FS) [4], RLA rats showed a reactive coping behavior mainly characterized by freezing, grooming, and tail licking, whereas RHA rats displayed a strong proactive coping behavior, spending a longer time biting the clamp to remove it from their tails. These results confirmed and extended those of our previous behavioral studies [5,6,14,18], showing that, when exposed to an acute and severe stressor such as FS, RLA rats exhibited a reactive coping behavior compared to their RHA counterparts. 

### 3.1. Effect of Tail Pinch-Induced Acute Stress on the BDNF and trkB Protein Levels in the Dorsal and Ventral Hippocampus

Together, the densitometric analyses of the WBs of hippocampal tissue homogenates showed that the basal levels of both BDNF and trkB are lower in the vHC of RLA than RHA rats. Our findings further indicated that a stress modality as mild as a 40 min TP may interfere with the basal BDNF/trkB signaling in both lines. Interestingly, the effects of TP are only partially congruous with those obtained upon FS [18]. Specifically, both stress modalities affect the hippocampal expression of BDNF and trkB, with differences between the dHC and the vHC that fit with the role and connectivity displayed along the longitudinal septo-temporal axis of the HC [27,28]. Thus, the dHC, which corresponds to the posterior HC of primates, performs primarily cognitive functions while the vHC (anterior HC in primates) relates to stress, anxiety, fear and reward seeking [28]. Importantly, the vHC plays an important role sorting the appropriate behavioral response (e.g., avoidance or freezing) to situations of conflict, such as the typical approach-avoidance condition during the acquisition of the active avoidance task that was used as the selection criterion of the Roman lines [29,30]. 

Our present results report on the TP-induced BDNF protein changes at a single time point following the beginning of the stress. However, recent evidence shows that, once the tested animal has perceived the post-encounter danger, the vHC, acting as an arbitrator-comparator, plays a key role driving the type of peripheral systemic response to environmental cues [31] by either releasing or blocking the behavior controlled by the hypothalamus [32]. 

Moreover, our results are consistent with ample evidence suggesting that a dynamic and rapid regulation of the BDNF expression and signaling is implicated in the effect of acute stress on the hippocampal structure and connectivity [33,34,35,36,37,38]. 

The increase in BDNF protein levels observed in the dHC of RLA rats upon both TP and FS is in keeping with the increment in the BDNF mRNA or protein levels caused by different types of acute stress [39,40,41,42] and may be interpreted as an adaptive neuronal plasticity response to the aversive novelty. However, in RHA rats, TP, which induces a proactive behavior aimed at removing the clamp from the tail but not anxiety-like behaviors [4 and present study], is also associated with alterations in BDNF signaling that are not present upon FS [18]. Thus, in the dHC, the increment of protein levels upon TP affected not only BDNF but also trkB levels and involved both Roman lines. At present, there is no clear explanation for these unexpected findings that deserve further investigation.

In the vHC, TP induced a decrease in BDNF protein levels in RHA rats and trkB in RLA rats. This result contrasts with the lack of effect of FS on BDNF/trkB signaling in the vHC of RHA rats, since the BDNF protein levels observed in our previous study, although tendentially reduced, did not reach statistical significance [18]. However, as discussed afterwards, when considering the statistical analysis of the immunohistochemical densitometric values, both FS [18] and TP [present data] elicit a reduction of BDNF/trkB signaling in the vHC subregions of RHA rats. Moreover, FS, but not TP, elicited a marked decrease in the levels of BDNF in the vHC of RLA rats [18]. Therefore, it is plausible that an intense stressor like FS, but not a mild stressor like TP, may hinder neuroplastic events in the vHC of RLA but not RHA rats [18]. 

Collectively, the above results further corroborate the notion that a mild acute stressor also impacts the BDNF and trkB protein levels thereby inducing fast neuroplastic adaptations that may prove useful to optimize the animal’s coping ability. 

### 3.2. Effect of Tail Pinch on the Regional and Subregional Immunohistochemical Distribution of BDNF and trkB in the Dorsal and Ventral Hippocampus

TP induced distinct changes in BDNF-LI and trkB-LI across the Roman lines and along the septo-temporal extension of the HC. Thus, the densitometric analysis of the immunostained tissue sections revealed that, in the dHC, RLA rats displayed an increment over the basal level of BDNF-LI in the CA2 and CA3 sectors of the Ammon’s horn, whereas RHA rats showed an increment over the basal level of BDNF-LI only in the CA2 sector. Regarding trkB-LI, RHA rats displayed a small albeit significant increment over the basal value in the DG, while RLA rats showed no significant changes. In the vHC, RHA rats showed a decrease in the basal BDNF-LI in the CA1 sector of the Ammon’s horn, whereas in RLA rats, no significant differences occurred. As for trkB-LI, RHA rats displayed a small increment over the basal value in the DG of the dHC and a decrement in the CA1 sector of the vHC; in contrast, RLA rats showed no significant changes either in the dHC or the vHC. 

All in all, the results of the IHC assays are consistent with those of the densitometric analyses of the WBs, although the IHC assays failed to reveal a significant decrease in trkB-LI in the vHC of RLA rats (compare Figure 1 and Figure 2 with Figure 5, Figure 6, Figure 9 and Figure 10). 

It is noteworthy, however, that when comparing the TP- with the FS-induced changes in the ligand and receptor protein levels of the dHC of RHA rats, the TP-induced increments of BDNF-LI in the CA2 and trkB-LI in the DG are not present upon FS [18], suggesting that they may represent molecular adjustments to support the proactive coping of RHA rats in the face of a mild stressor but not a severe stressor. In contrast, in the vHC of RLA rats, the region where, upon FS, both the BDNF- and trkB-LI decreased markedly, TP fails to induce any changes. As regards the RHA line, the decrease in BDNF- and trkB-LI in the vHC tissue sections after TP reaches a statistical significance only in the CA1 sector, while upon FS the density of BDNF- and trkB-immunoreactivities are reduced in the whole vHC and in the CA1 and DG, respectively. In the rat, the CA1 sector is known to play a role as a ‘novelty’ detector that identifies the mismatches between the set of inputs from the entorhinal cortex, concerning the current situation, and those from the CA3, concerning the stored predictions [33]. Whether the BDNF/trkB changes observed in the CA1 may account for the different coping responses of RHA vs. RLA rats is an issue worth further investigation. Together, these findings indicate that the impact on BDNF/trkB signaling of a mild stressor like TP is mediated, at least in part, through different cellular pathways than those involved in the effects of an intense stressor like FS [43]. Much work remains to be done to identify such different signaling pathways. 

The CA3 subfield is subjected dynamically to a process of continuous adjustment of its connectivity due to a persistent invasion of new mossy fiber projections along with the formation of new synaptic contacts and contextual growth and retraction of the pyramidal dendritic arborizations [41,42,43,44]. On the other hand, the CA3 sector has been shown to act as an autoassociation system of the information arriving from different cortical areas [33], with the perforant pathway from the entorhinal cortex playing an important role in the retrieval of information.

Therefore, given the increments in BDNF-LI in the CA3 sector of the dHC, it may be hypothesized that BDNF/trkB signaling is involved in the modulation of TP-induced plastic events in the dHC of both Roman lines. 

We previously noted that the lower levels of BDNF protein in the CA3 subfield of the dHC of control RLA vs. RHA rats could be due to a slower synthesis rate of neurotrophin in that region [13]. BDNF is both locally produced and anterogradely transported along the mossy fibers in the CA3 sector. Hence, according to the neurotrophic hypothesis of depression, the presumably slower production of BDNF protein in RLA rats may, in turn, lead to a deficit in the synaptic release of BDNF and reduced target-derived support to promote the synaptic contacts with the mossy fibers. In fact, besides the potential autocrine/paracrine effects within the granule cell population, BDNF potently regulates the synaptic plasticity of mossy fibers. Accordingly, the increased expression of BDNF in the forebrain of transgenic mice stimulates the sprouting of mossy fibers, expands their innervation of the CA3 stratum oriens, and regulates the extension of their infrapyramidal and suprapyramidal projections [45]. 

### 3.3. Insights into the Possible Role of Stress Hormones in the Effects of Tail Pinch on BDNF/trkB Signaling in the Dorsal and Ventral Hippocampus

Over the past decades, several research groups have formulated a hypothesis relating alterations in cortisol, corticotrophin (ACTH), and corticotrophin-releasing hormone (CRH) functions to the causality of depression and proposed that antidepressants may act through normalization of the hypothalamus-pituitary-adrenal axis (HPA) dysfunctions [46]. 

Experimental support for this hypothesis was provided by neuroendocrine function measurements, including the dexamethasone/CRH (dex/CRH) test [47]. In this test, patients are pretreated with dexamethasone at 23:00 h and, the next day, receive CRH at 15:00 h. The amount of ACTH and cortisol subsequently released is much higher in a subpopulation of depressives, confirming the close association between HPA dysregulation and depressive psychopathology [48]. 

Notably, the functional characteristics of the HPA axis of RLA rats are reminiscent of those of depressives during a Major Depression Episode (MDD) [47]. Thus, the characterization of the stress response of Roman rats confronted with adverse stimuli consistently demonstrates that exposure to minor stressful events causes a transient elevation of plasma corticosterone levels in both lines, with more pronounced amplitude and duration in RLA vs. RHA rats [49,50,51,52]. Most important, in the dex/CRH test the increment of plasma corticosterone concentration is significantly higher in RLA vs. RHA rats [26,53]. 

Corticosterone binds to two receptor types, the high-affinity mineralocorticosteroid receptors (MRs) and the low-affinity glucocorticosteroid receptors (GRs) [27,54,55]. Remarkably, the density of corticosteroid receptors is lower in the HC of RLA vs. RHA rats [56].

The dHC and vHC have been shown to distinctly differ in the density and balance of MRs and GRs [27,56,57] and their stress-related dynamic connectivity [54,58], with the vHC expressing more MRs and the dHC containing more GRs [59,60]. 

Several data evidenced the close relationship between stress hormone levels and modulation of BDNF and trkB levels. Thus, early studies carried out in the dHC with corticosterone administration and 4 to 6 h of follow-up showed that BDNF mRNA levels decreased with increasing doses, while trkB mRNA levels increased with higher doses of corticosterone [61]. Furthermore, the acute administration of glucocorticoids has been shown to promote trkB activation in the dHC in vivo [62,63] without an increase in BDNF production [62]. Therefore, the distinct functional effects of stress hormones in the dHC and vHC [27,64,65], together with the different densities of hippocampal corticosteroid receptors across the Roman lines may account, at least in part, for the different impact of TP on BDNF and trkB protein expression along the hippocampal longitudinal axis of RLA vs. RHA rats. Moreover, activation of the MAPK kinase signaling cascade by trkB, associated with the deactivation of a GR-protein phosphatase 5 pathway leads to a sustained GR phosphorylation at BDNF-sensitive sites that is essential for the transcription of genes involved in neuronal plasticity [66].

Notably, the disruption of GR phosphorylation or trkB signaling in vivo impairs the neuroplastic effects of chronic stress and the response to the antidepressant fluoxetine [66]. This finding supports the view that the coordinated signaling of BDNF/trkB and glucocorticoids promote neuronal plasticity and that disruption in either pathway could set the stage for the development of stress-induced psychiatric diseases. Therefore, the higher increment in corticosterone levels elicited by a variety of stressors in RLA vs. RHA rats may account, at least in part, for the different effects of TP on the levels of BDNF and trkB in the hippocampal subfields of the Roman lines.

## 4. Materials and Methods

### 4.1. Animals

Male outbred Roman rats (N = 28 for each line) were used throughout and were four months old (weight = 400–450 g) at the beginning of the experiments.

The animals were housed in groups of four per cage and maintained under temperature- and humidity-controlled environmental conditions (23 °C ± 1 °C and 60% ± 10%, respectively), with a 12 h light–dark cycle (lights on at 8:00 a.m.). Standard laboratory food and water were available *ad libitum*. To avoid stressful stimuli resulting from manipulation, the maintenance activities in the animal house were carried out by a single attendant and bedding in the home cages was not changed on the two days preceding the test. All procedures were performed according to the guidelines and protocols of the European Union (Directive 2010/63/EU). The experimental protocol was approved by the Committee for Animal Experimentation of the Universidad Autónoma de Barcelona and was authorized by the Ministerio de Ciencia e Innovación (authorization No. PID2020-114697GB-I00, 1 January 2021). Every possible effort was made to minimize animal pain and discomfort and to reduce the number of experimental subjects.

### 4.2. TP and Behavioral Measurements

RHA and RLA rats (N = 28 for each line, naïve at the beginning of the experiments) were randomly assigned to the control or TP groups and were processed in parallel, according to a schedule that was counterbalanced for animal line. Rats in the TP groups (N = 14 for each line) were moved singly from the animal house to an adjacent sound-attenuated, dimly illuminated test room whereas the controls (N = 14 for each line) were kept in their home cages in the animal house until sacrifice. All testing was performed between 10:00 a.m. and 6:00 p.m. and the experimental conditions were as previously described [4]. Rats in the TP groups were placed individually in a makrolon cage (40 cm × 28 cm × 20 cm) with sawdust bedding, and their tails were introduced through a rubber tubing 20 mm in length (i.d. 5 mm, 1 mm thick). The tubing was positioned at 6 cm from the tip of the rats’ tails and the clamp was delicately tightened around the tubing for 40 min to induce discomfort and arousal, but not pain [4,19].

Along the 40 min in which the clamp tightened the rat’s tail a single well-trained observer that was blind to the rat line recorded the behaviors occurring during 5 s time windows at 1 min intervals. The following behaviors were recorded: (1) biting the clamp to loosen and remove it from the tail, (2) licking the tail near the clamp, (3) freezing, characterized by complete immobility, including the vibrissae, and with the exception of respiratory movements, and (4) grooming of the head with the forelimbs, of the body with the head, or of the body with the hindlimbs. The behaviors were recorded on pre-prepared checklists, according to the time-sampling method described above, and the total time each rat was engaged in any of the behaviors listed above throughout the TP session was recorded and used for statistical analysis.

At the end of the TP session, rats were removed from the makrolon cages and transferred singly to an adjacent room where they were sacrificed. The behaviors were recorded only in a representative sample of animals from the control and TP groups that were subsequently used for the Western blot (eight rats from each line) or immunohistochemical assays (six rats from each line).

### 4.3. Sampling

Immediately after the end of the TP session, the animals used for the WBs (N = 32) were killed by decapitation whereas the animals used for the immunohistochemical assays (N = 24) were deeply anesthetized with chloral hydrate (500 mg/kg, i.p., 2 mL/kg) and a few minutes later were transcardially-perfused with ice-cold PBS (Phosphate Buffered Saline: 137 mM NaCl, 2.7 mM KCl, 10 mM Na_2_HPO_4_, 2 mM KH_2_ PO_4_, pH 7.3) and 4% paraformaldehyde.

**Figure 11:**
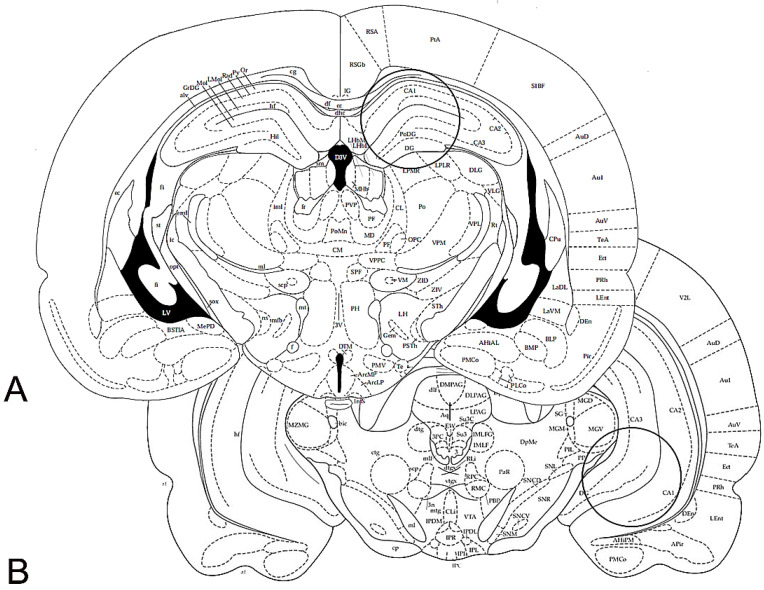
Schematic representation of two rat brain coronal sections (Figure 33 and Figure 44, modified from Paxinos and Watson [25]). The circles denote the areas of the dorsal (**A**) and ventral (**B**) hippocampus, taken for Western blot analysis, by means of a 2.5 mm punch. Stereotaxic coordinates (from Bregma): (**A**) −3.30 mm, (**B**) −6.04 mm.

The brains were rapidly removed from the skull immediately after sacrifice for the WB assays or after perfusion for the IHC experiments. For WB, the brains were cooled in dry ice for 15 s, placed in a brain matrix, and cut in 2 mm thick coronal slices, using the stereotaxic coordinates of the rat brain atlas of Paxinos and Watson [25] as a reference. The anterior-posterior coordinates (from bregma) were approximately −3.30 mm and −6.04 mm, for the dorsal and vHC, respectively. Bilateral punches (diameter 2.5 mm) of the dHC and vHC were taken, as described by Palkovits [67] (Figure 11), The tissue punches were rapidly frozen at −80 °C and homogenized in distilled water containing 2% sodium dodecyl sulfate (SDS) (300 μL/100 mg of tissue) and a cocktail of protease inhibitors (cOmpleteTM, Mini Protease Inhibitor Cocktail Tablets, Cat# 11697498001, Roche, Basel, Switzerland). For IHC, the brains were post-fixed by immersion in a freshly prepared 4% phosphate-buffered PFA, pH 7.3, for 4–6 h at 4 °C, and then rinsed until they sank in 0.1 M phosphate buffer (PB), pH 7.3, containing 20% sucrose.

### 4.4. Western Blot

Total protein concentrations were determined as described by Lowry et al. [68], using bovine serum albumin as a standard. Proteins from each tissue homogenate (40 μg), diluted 3:1 in 4× loading buffer (NuPAGE LDS Sample Buffer 4×, Cat# NP0008, Novex by Life Technologies, Carlsbad, CA, USA), were heated to 95 °C for 7 min, and separated by sodium dodecyl sulfate (SDS)-polyacrylamide gel electrophoresis (SDS-PAGE), using precast polyacrylamide gradient gel (NuPAGE 4–12% Bis-Tris Gel Midi, Cat# NP0321, Novex by Life Technologies, Carlsbad, CA, USA), in the XCell4 Sure LockTM Midi-Cell chamber (Life Technologies, Carlsbad, CA, USA). Internal mw. standards (Precision Plus Protein Western C Standards, Cat# 161-0376, Bio-Rad, Hercules, CA, USA) were run in parallel. Blots were blocked by immersion in 20 mM Tris base and 137 mM sodium chloride (TBS), containing 0.1% Tween 20 (TBS-T) and 5% milk powder, for 60 min, at room temperature. The primary antibodies were rabbit polyclonal antibodies against BDNF (Cat# N-20 sc-546, RRID: AB_630940, Santa Cruz Biotechnology, Dallas, TX, USA) and trkB (Cat# (794) sc-12, RRID: AB_632557, Santa Cruz Biotechnology, Dallas, TX, USA), both diluted 1:1000 in TBS containing 5% milk powder and 0.02% sodium azide. Incubations with primary antiserum were carried out for two nights at 4 °C. After rinsing in TBS/T, blots were incubated at room temperature, for 60 min, with a peroxidase-conjugated goat anti-rabbit serum (Cat#9169, RRID:AB_258434, Sigma Aldrich, St Louis, MO, USA), diluted 1:10,000 in TBS/T. Controls for equal-loading of the wells were obtained by immunostaining the membranes, as above, using a mouse monoclonal antibody against glyceraldehyde-3-phosphate dehydrogenase (GAPDH) (MAB374, RRID:AB_2107445, EMD Millipore, Darmstadt, Germany), diluted 1:1000, as the primary antiserum, and a peroxidase-conjugated goat anti-mouse serum (AP124P, RRID:AB_90456, Millipore, Darmstadt, Germany), diluted 1:5000, as the secondary antiserum. To control for non-specific staining, blots were stripped and incubated with the relevant secondary antiserum. In order to check for antibody specificity and cross-reactivity, the anti-BDNF antibody was challenged with 200 ng of rhBDNF (Cat# B-257, Alomone Labs, Jerusalem, Israel) [13]. After rinsing in TBS/T, protein bands were developed using the Western Lightning Plus ECL (Cat# 103001EA, PerkinElmer, Waltham, MA, USA), according to the protocol provided by the manufacturer, and visualized using the ImageQuant LAS-4000 (GE Healthcare, Little Chalfont, UK). Approximate molecular weight (mw) and relative optical density (O.D.) of the labeled protein bands were evaluated by a blinded examiner. The ratio of the intensity of the BDNF-positive and trkB-positive bands to the intensity of the GAPDH-positive ones was used to compare the relative expression levels of these proteins in both rat lines. The O.D. was quantified by the Image Studio Lite Software (RRID:SCR_014211, Li-Cor).

### 4.5. Immunohistochemistry

Coronal brain sections from the RLA and RHA rats were examined in pairs placed on the same slide. Semiconsecutive cryostat sections (14 μm thick) were collected on chrome alum-gelatin coated slides and processed by the avidin–biotin–peroxidase complex (ABC) immunohistochemical technique. The endogenous peroxidase activity was blocked with 0.1% phenylhydrazine (Cat# 101326606, Sigma Aldrich, St Louis, MO, USA) in phosphate-buffered saline (PBS), containing 0.2% Triton X-100 (PBS/T), followed by incubation with 20% of normal goat serum (Cat# S-1000, Vector, Burlingame, CA, USA). The primary antibodies (i.e., rabbit polyclonal antibodies against BDNF and trkB) were the same as those used for WB and both were diluted 1:500. A biotin-conjugated goat anti-rabbit serum (BA-1000, RRID: AB_2313606, Vector, Burlingame, CA, USA), diluted 1:400, was used as a secondary antiserum. The reaction product was revealed with the ABC (Cat#G011-61, BioSpa Div. Milan, Italy), diluted 1:250, followed by incubation with a solution of 0.1 M PB, pH 7.3, containing 0.05% 3,3′-diaminobenzidine (Sigma Aldrich, St Louis, MO, USA), 0.04% nickel ammonium sulfate and 0.01% hydrogen peroxide. All antisera and the ABC were diluted in PBS/T. Incubation with primary antibodies was carried out overnight at 4 °C. Incubations with the secondary antiserum and ABC lasted 60 min and were performed at room temperature. Negative control preparations were obtained by incubating tissue sections in parallel with either PBS/T, alone, or in one of the following ways: (i) with the relevant primary antiserum pre-absorbed with an excess of the corresponding peptide antigen (Cat# sc-546P and sc-12 P, for BDNF and trkB, respectively, Santa Cruz Biotechnology, Santa Cruz, CA, USA), or (ii) by substituting the corresponding primary antiserum with normal goat serum. Slides were observed with an Olympus BX61 microscope and digital images were captured with a Leica DFC450C camera.

### 4.6. Image Densitometry

For a semiquantitative evaluation of the BDNF and the trkB immunohistochemical labeling, representative 10× magnification microscopic fields were taken from twelve coronal sections of six animals for each experimental group. The sections corresponded, approximately, to the AP coordinates used to obtain the tissue samples used for the WB assays, and were blindly analyzed with ImageJ (http://rsb.info.nih.gov/ij/; RRID:SCR_003070) to calculate the density of immunoreactivity per µm^2^. Briefly, images of the subregions of the dHC and vHC (i.e., CA subfields and DG) were captured according to a standardized framing for each subregion and thresholds were blindly established to a set level to reveal the BDNF- and trkB-immunolabeling. The maximum threshold value was adjusted so that the background signal could be subtracted without removing the true immunoreactive signal. The relative O.D. of immunoreactivity was automatically calculated as the ratio of the area occupied by the immunoreactive structures to the area of the image file. Mean gray values from the unstained areas were subtracted from the gray values of the immunostained regions, to exclude the background staining.

### 4.7. Statistical Analyses

Behavioral measurements were statistically evaluated using the Student’s *t* test for independent samples. WB and immunohistochemical data were statistically evaluated by two-way ANOVA (see Tables S1 and S2). Before performing both Student’s *t* tests and the ANOVAs, data sets of each experimental condition were inspected for normal distribution of data and homogeneity of variances, with the Shapiro–Wilk test and the Bartlett test, respectively. Among the behavioral measurements, the diving dataset showed statistically significant unequal variances and, therefore, were analyzed with the Welch *t* test. Data sets that did not show homogeneity of variances were log-transformed, and then analyzed by two-way ANOVA, as previously described [14,18,69]. When two-way ANOVAs revealed statistically significant interactions, the sources of significance were ascertained by pairwise *post-hoc* contrasts with the HSD Tukey test. In all the other cases, pairwise comparisons were performed with the two-tailed *t* test with Sidak’s corrected alpha values. All the statistical analyses were carried out with the PRISM, GraphPad 6 Software (San Diego, CA, USA) with the significance level set at *p* < 0.05.

## 5. Conclusions

In this study, we provide experimental evidence based on WB and IHC assays demonstrating that a very mild acute stressor like TP elicits rapid and distinct changes in the levels of BDNF and trkB proteins in different hippocampal subfields of RHA and RLA rats. 

The comparison between the results of the present study and those of our previous work with FS [18] suggests that the intensity of the stressor strongly influences the modifications induced on hippocampal BDNF/trkB signaling in a line-dependent manner. However, the molecular mechanisms underlying the distinct effects of TP on BDNF/trkB signaling in RLA vs. RHA rats remain elusive. Of note, the concomitant activity of neurotrophins and glucocorticoids elicits robust modifications of synaptic structure and function, and alterations of their activities are a risk factor for vulnerability to stress-related disorders. Thus, low BDNF levels increase the desensitization of GRs and hence the vulnerability to stress, whereas higher levels of BDNF facilitate both GR-mediated signaling and the response to antidepressants [70]. Interestingly, an association between BDNF and cortisol levels has been also reported in healthy humans during acute psychosocial stress [70,71]. It has been found that the stress-induced BDNF peak is associated with a swift recovery of cortisol resting levels [70], an event that appears crucial to prevent sustained activation of the HPA axis and, thus, is highly adaptive in reducing the vulnerability to stress-induced pathologies [70,71,72]. Collectively, these findings suggest that the coordinated actions of BDNF and glucocorticoids promote neuronal plasticity and that disruption in either pathway may lead to stress-induced psychiatric disorders [66].

Further studies in Roman rats aimed at identifying BDNF-related intracellular pathways and possibly different mutated variants of this trophin and its trkB receptor are warranted to shed light on the mechanisms promoting resilience or vulnerability under adverse situations.

## Figures and Tables

**Table 1:** Behaviors displayed by RHA and RLA rats during the tail pinch session.

Behavioral Measures (s)	RHA	RLA
Clamp biting	789 ± 60	294 ± 51 ****
Tail licking	161 ± 23	326 ± 34 ***
Freezing	5 ± 2	134 ± 17 ****
Grooming	43 ± 5	81 ± 11 *

Shown are the mean ± SEM of eight rats in each experimental group. * *p* < 0.05; *** *p* < 0.001; ****: *p* < 0.0001 vs. the RHA group (two-tailed Student’s *t* test for independent samples).

## Data Availability

The data presented in the current study are available from the corresponding author on reasonable request.

## Supplementary Materials

The following supporting information can be downloaded at: https://www.mdpi.com/article/10.3390/ijms24119498/s1.

## Abbreviations

ABC	Avidin–Biotin–peroxidase Complex
BDNF	Brain Derived Neurotrophic Factor
DG	Dentate Gyrus
dHC	dorsal Hippocampus
FS	Forced Swimming
GAPDH	Glyceraldehyde-3-Phosphate Dehydrogenase
HPA	Hypothalamus–Pituitary–Adrenal
IHC	Immunohistochemistry
O.D.	Relative optical density
PBS	Phosphate Buffered Saline
PFC	Prefrontal cortex
RHA	Roman High-Avoidance
RLA	Roman Low-Avoidance
SDS-PAGE	Sodium dodecyl sulphate-polyacrylamide gel electrophoresis
TBS-T	Tris base, Sodium chloride, Tween 2
TP	Tail Pinch
trkB	tyrosine receptor kinase B
vHC	ventral Hippocampus
VTA	Ventral Tegmental Area
WB	Western Blot
