# Supplementary material for: Effects of Tail Pinch on BDNF and trkB Expression in the Hippocampus of Roman Low- (RLA) and High-Avoidance (RHA) Rats
Source: Int J Mol Sci. 2023 May 30;24(11):9498. doi: 10.3390/ijms24119498 (PMC10253481; doi:10.3390/ijms24119498)
Supplement: Supplementary file 1 [file ijms-24-09498-s001.zip › ijms-2394963-supplementary.pdf]

Article

Effects of Tail Pinch on BDNF and trkB Expression in the Hippocampus of Roman Low- (RLA) and High-Avoidance (RHA) rats

Maria Pina Serra [1,†], Francesco Sanna [2,†], Marianna Boi [1], Marcello Trucas [1], Alberto Fernández-Teruel [3], Maria Giuseppa Corda [2], Osvaldo Giorgi [2,‡] and Marina Quartu [1,*,‡]

1 Department of Biomedical Sciences, Section of Cytomorphology, University of Cagliari, Cittadella Universitaria di Monserrato, 09042 Monserrato (CA), Italy; marianna.boi@unica.it (M.B.); marcello.trucas@unica.it (M.T.)
2 Department of Life and Environmental Sciences, Section of Pharmaceutical, Pharmacological and Nutraceutical Sciences, University of Cagliari, Cittadella Universitaria di Monserrato, 09042 Monserrato, Italy
3 Medical Psychology Unit, Department of Psychiatry and Legal Medicine & Institute of Neurosciences, School of Medicine, Autonomous University of Barcelona, Bellaterra, 08193 Barcelona, Spain
* Correspondence: quartu@unica.it; Tel.: +39-070-675-4084
† These authors contributed equally to this work (joint first authors).
‡ These authors contributed equally to this work (joint senior authors).

Table S1. F values and significance levels of two-way ANOVAs performed on western blot data, shown in Figures 1 and 2.

Brain Area	Marker	Line		TP		Line × TP		
		F	*p*	F	*p*	F	*p*	*d.f.*
Dorsal Hippocampus	BDNF	5.506	0.0263	28.74	<0.0001	0.1175	ns	1.28
	trkB	0.04214	ns	26,69	ns	1,230	<0.0001	1.28
Ventral Hippocampus	BDNF	8.730	0.0063	13.68	0.0009	3.438	ns	1.28
	trkB	7.049	0.0129	1.845	0.1852	6.271	0.0184	1.28

ns—not significant; *d.f.*—degrees of freedom.

Table S2. F values and significance levels of two-way ANOVAs performed on data obtained from the densitometric analysis of tissue section distribution of BDNF-like immunoreactivity (LI) and trkB-LI shown in Figures 1–4.

Brain Area	Marker	Line		TP		Line × TP		
		F	*p*	F	*p*	F	*p*	*d.f.*
Dorsal Hippocampus								
CA1	BDNF	3.834	0.0566	3.963	0.0527	0.9296	ns	1.44
	trkB	6.169	0.0169	0.05298	ns	0.6357	ns	1.44
CA2	BDNF	61.03	<0.0001	29.13	<0.0001	1.023	ns	1.44
	trkB	0.5791	ns	0.1592	ns	0.7378	ns	1.44
CA3	BDNF	51.66	<0.0001	33.83	<0.0001	16.5	0.0002	1.44
	trkB	0.9633	ns	0.02472	ns	0.08183	ns	1.44
DG	BDNF	10.7	0.0021	4.792	0.0339	1.591	ns	1.44
	trkB	6.941	0.0116	5.439	0.0243	10.90	0.0019	1.44
Ventral Hippocampus								
CA1	BDNF	5.148	0.0282	6.545	0.0140	5.208	0.0274	1.44
	trkB	8.681	0.0051	2.445	ns	7.378	0.0094	1.44

CA3	BDNF	1.440	ns	0.04668	ns	0.01355	ns	1.44
	trkB	28.08	<0.0001	0.01347	ns	0.01355	ns	1.44
DG	BDNF	0.2088	ns	1.780	ns	0.5457	ns	1.44
	trkB	18.29	0.0001	5.391	0.0249	0.03890	ns	1.44

ns—not significant; *d.f.*—degrees of freedom.
